# Blood Transfusion Predictors in Cesarean Sections for Pregnancies With Placenta Accreta and Placenta Previa: A Monocentric Tertiary Experience

**DOI:** 10.7759/cureus.47648

**Published:** 2023-10-25

**Authors:** Zainab G Alhashim, Zainab A Alzayer, Alwi A Alensaif, Hussain A Al Darwish, Mohammed A Almomen, Jawad M Alnsaif

**Affiliations:** 1 Department of Anesthesiology, Imam Abdulrahman Bin Faisal University College of Medicine, Dammam, SAU; 2 Department of Anesthesiology, King Fahad University Hospital, Al-Khobar, SAU; 3 College of Medicine, Imam Abdulrahman Bin Faisal University College of Medicine, Dammam, SAU

**Keywords:** risk factors, placenta accreta, placenta previa, cesarean section, blood transfusion

## Abstract

Objective

The objective of this study was to analyze the possible predictors of the need for intraoperative blood transfusion in cesarean sections for pregnancies with abnormal placentation.

Methods

This was a retrospective study based on data from patients’ electronic medical records. A total of 44 patients who were diagnosed as placenta previa or placenta accreta who delivered through cesarean section at King Fahad University Hospital, Al-Khobar, Saudi Arabia, from June 1997 to January 2021 were included in the study. Seventeen patients received intra-operative blood transfusion. The other 27 patients did not receive any blood transfusions and served as controls. Demographic data, antepartum profiles, and obstetric history were compared between the two groups. Univariate analysis and multivariate logistic regression were used to analyze the correlations between related risk factors and the need for intraoperative blood transfusion.

Results

Univariate analysis (χ2 test) has shown multiple factors that correlated significantly (p<0.05) with blood transfusion requirement. These factors include the presence of placenta accreta, general anesthesia, preoperative hematocrit < 33%, preoperative hemoglobin ≤ 10 g/dL, and preterm delivery at 35-36 weeks of gestation. None of these factors showed any statistical significance in multivariate analysis (logistic regression).

Conclusion

General anesthesia, placenta accreta, delivery at 35-36 weeks of gestation, and pre-operative anemia are possible risk factors for blood transfusion during cesarean sections for abnormal placentation. Identifying patients at increased risk is necessary to optimize pre-operative and intraoperative management.

## Introduction

Abnormal placentation incorporates multiple conditions including placenta previa and placenta accreta spectrum (PAS). Placenta previa can be defined as placental tissue that completely or partially covers the internal cervical os. PAS is a condition that encompasses the types of morbid placenta adherence. Depending on the depth of villous invasion, PAS could be classified into accreta, increta, or percreta. Placental disorders are well-established risk factors for serious fetal and maternal morbidity, and even mortality [1].

In conjunction with the rise of cesarean deliveries, a surge of abnormal placentation cases is noted, leading to increasing attention to these conditions in recent years [2,3]. The rapid increase in the prevalence of placentation disorders is associated with higher levels of complications such as hysterectomy, peripartum hemorrhage, and mortality. Placenta previa and PAS are among the leading causes of peripartum hemorrhage and peripartum hysterectomy [1,3,4]. Though the prevalence of abnormal placentation is not accurately documented, a rough estimate can be inferred through a literature review. Earlier reports estimated prevalence to be as low as 1/2000. In more recent studies, prevalence estimations are reaching up to 1/500 [5]. As a tertiary center to which cases of abnormal placental implantations are referred, an increased rate of reference has been noticed.

As mentioned, abnormal placentation disorders are among the leading etiologies for peripartum hemorrhage and hysterectomy. Hysterectomy is the most commonly performed gynecological procedure in the United States [6]. Other than the psychological impact of losing fertility, the procedure is associated with various complications such as infections, mechanical injuries to the genitourinary tract or bowel, neuropathies, vaginal cuff dehiscence, and maternal mortality [7,8]. Intraoperative bleeding and its sequelae represent a major risk factor for morbidity and mortality in these patients, and the volume of blood loss could be used as a predictor of the outcome [9-12].

In the literature, a variation in blood loss volumes between cases of abnormal placentation cesarean sections is noted [9-12]. Further research regarding risk stratification and management of intraoperative blood loss in these cases is needed. Within the current body of literature, only a few articles have discussed the relation of possible risk factors to intraoperative bleeding risk in these conditions. Therefore, this study was conducted to analyze these risk factors and hopefully aid clinicians in identifying patients with an increased risk of bleeding. Consequently, improving perioperative and intraoperative management and outcomes. 

## Materials and methods

This retrospective case-control study was conducted after approval by the Institutional Review Board at King Fahad University Hospital (approval number: RB-UGS-2022-01-423). Permission to extract clinical data through the medical electronic record system (QuadraMed; QuadraMed Corporation, Reston, Virginia, United States) was provided by the director of the Department of Anesthesiology.

A total of 110 patients diagnosed with placenta previa and, or placenta accreta who underwent cesarean section at King Fahad University Hospital, Al-Khobar, Saudi Arabia, between June 1997 and January 2021, were initially included in this study. From this, 66 patients had to be excluded due to incomplete documentation of their preoperative and intraoperative data. A total of 44 subjects were thus included in the final dataset of this study.

The case group (n=17) was composed of patients who received a minimum of one unit of blood during their cesarean delivery. The control group (n=27) compromised patients who did not receive any blood products during the operation.

Electronic medical data were carefully extracted and compared between both groups. Data collected specifically included maternal demographical data, antepartum profile, obstetric history, and preoperative and postoperative laboratory investigations. Additionally, operative data and operative outcomes and complications were collected and analyzed.

Statistical analysis

Univariate (χ2 test) and multivariate (logistic regression) analyses were performed using GraphPad Prism software version 9.5.1 (Dotmatics, Boston, Massachusetts, United States). Prior to conducting the univariate analysis, variables were subdivided into subcategories of two or three. Significant variables identified via the univariate analyses (p < 0.05) were then enrolled into the multivariate analysis to reach a final model of independent predicting factors of blood transfusion.

## Results

This study included 44 subjects, of which 27 (61%) comprised the non-transfusion group and 17 (39%) were included in the transfusion group. The study compared multiple factors between the two groups including patient demographics, obstetric history, placental features, types of anesthesia, and surgical course, which are presented in Table 1.

**Table 1:** Univariate Analysis (χ2 test) OR: odds ratio; CL: confidence interval; APH: antepartum haemorrhage

Predicting factors					Univariate analysis	
Parameter	Transfusion	(%)	No transfusion	(%)	chi square, do	P-value
Total 44	17		27			
Demographic data						
Maternal Age (years)						
< 35	5	29.41%	11	40.74%	0.5786, 1	0.4469
≥ 35	12	70.59%	16	59.26%		
Gestational Age (weeks)						
27-34	5	29.4%	10	37.0%	7.562,2	0.0228
35-36	7	41.2%	2	7.4%		
≥ 37	5	29.4%	15	55.6%		
Gravidity						
≤ 2	2	11.76%	9	33.33%	2.588,1	0.1077
>2	15	88.24%	18	66.67%		
Parity						
0	0	0.00%	4	14.81%	2.770,1	0.096
1	17	100.00%	23	85.19%		
Abortions						
No	9	52.94%	19	70.37%	1,369,1	0.2419
Yes	8	47.06%	8	29.63%		
Antepartum profile						
Number of Fetuses						
Singleton	15	88.24%	27	100.00%	3.328,1	0.0681
Multi	2	0.1176	0	0.00%		
Iron-Deficiency Anemia						
Yes	3	17.65%	4	14.81%	0.06255, 1	0.8025
No	14	82.35%	23	85.19%		
Hypertensive Conditions						
Pre-eclampsia/hypertension	2	11.76%	1	3.70%	1.067,1	0.3016
None	15	88.24%	26	96.30%		
Diabetic Conditions						
Gestational diabetes	3	17.65%	3	11.11%	0.3784,1	0.5385
None	14	82.35%	24	88.89%		
Preoperative Bleeding						
APH/hematuria	5	29.41%	4	14.81%	1.366,1	0.2425
None	12	70.59%	23	85.19%		
Antenatal Care Regularity						
Regular	13	76.47%	21	77.78%	0.01015, 1	0.9198
Irregular	4	23.53%	6	22.22%		
Placental Abnormality						
Previa	11	64.71%	26	96.30%	7.782, 1	0.0053
Previa and accreta	6	35.29%	1	3.70%		
Past obstetric history						
History of Previous Cesarean Section						
No	8	47.06%	18	66.67%	1.659, 1	0.1977
yes	9	52.94%	9	33.33%		
History of Previous Dilation Curettage						
Yes	1	5.88%	1	3.70%	0.1141, 1	0.7355
No	16	94.12%	26	96.30%		
History of Previous Myomectomy						
Yes	0	0.00%	1	3.70%	0.6443, 1	0.4222
No	17	100.00%	26	96.30%		
Previous Postpartum Hemorrhage						
Yes	3	17.65%	2	7.41%	1.086, 1	0.2974
No	14	82.35%	25	92.59%		
Operative factors						
Anesthesia Type						
General	15	93.75%	16	61.54%	5.316, 1	0.0211
spinal/regional/ combined	1	6.25%	10	38.46%		
Not documented	1		1		Excluded	
Tranexamic Acid						
Yes	3	17.65%	1	3.70%	2.454, 1	0.1172
No	14	82.35%	26	96.30%		
Laboratory results						
Preoperative Hemoglobin						
≥ 10 g/dl	8	47.06%	4	14.81%	5.468, 1	0.0194
>10 g/dl	9	52.94%	23	85.19%		
Preoperative Hematocrit – (Preoperative Anemia)						
< 33% (Yes)	13	76.47%	11	40.74%	5.371, 1	0.0205
≥ 33% (No)	4	23.53%	16	59.26%		
Preoperative Platelets - Preoperative Thrombocytopenia						
< 150 (Yes)	6	35.29%	4	15.38%	2.283, 1	0.1308
≥ 150 (No)	11	64.71%	22	84.62%		
Not documented	0		1		Excluded	

Univariate analysis (chi-square analysis) showed statically significant differences (p <0.05) in gestational age (p=0.0228), type of placental abnormality (p=0.0053), type of anesthesia provided (general vs. regional anesthesia) (p=0.0211), low pre-operative hematocrit (p=0.0205), and low pre-operative hemoglobin (p=0.0194).

The variables that were statically significant in the univariate analysis were then analyzed through multivariate analysis (logistic regression analysis) as shown in Table 2. Gestational age, type of placental abnormality, type of anesthesia, preoperative hematocrit, and hemoglobin were statistically insignificant when analyzed with logistic regression analysis.

**Table 2:** Multivariate Analysis (logistic regression) OR: odds ratio; CL: confidence interval

Predicting factors					Multivariate analysis	
Parameter	Transfusion	(%)	No transfusion	(%)	Adjusted OR (95% CL)	P-value
Total 44	17		27			
Gestational Age						
27-34 weeks	5	29.4%	10	37.0%	1.206 (0.49-3.15)	0.6848
35-36 weeks	7	41.2%	2	7.4%		
≥ 37 weeks	5	29.4%	15	55.6%		
Placental abnormality						
Previa	11	64.71%	26	96.30%	70.38 (2.71-33436)	0.0596
Previa and accreta	6	35.29%	1	3.70%		
Anesthesia type						
General	15	93.75%	16	61.54%	0.1422 (0.007-0.81)	0.0711
Spinal/ regional/ combined	1	6.25%	10	38.46%		
Laboratory						
Preoperative hemoglobin						
≥ 10 g/dl	8	47.06%	4	14.81%	0.4371 (0.05-3.09)	0.4118
>10 g/dl	9	52.94%	23	85.19%		
Preoperative hematocrit – (preoperative anemia)						
< 33% (Yes)	13	76.47%	11	40.74%	0.5161 (0.07-3.53)	0.4901
≥ 33% (No)	4	23.53%	16	59.26%		

This study has also compared operative and delivery outcomes between the two groups. The number of units transfused was also recorded, and it was seen that massive blood transfusions were carried out in six out of 17 patients in the transfusion group. In the transfusion group, 52% of the patients received one to two packed red blood cell units, while the other 47% received three or more units. Additionally, 41% required continued postoperative transfusion. Feto-maternal complications were also compared between the two groups. Five cases (29%) of the transfusion group experienced postpartum hemorrhage compared to one case (3%) in the non-transfusion group. Additionally, hysterectomy was performed in six cases (35%) of the transfusion group compared to two cases (7%) of the non-transfusion group. None of the patients within the non-transfusion group were admitted to the intensive care unit. On the other hand, the transfusion group had six (35%) intensive care unit admissions. APGAR score was collected and compared between the two groups (Table 3).

**Table 3:** Delivery outcomes and complications ICU: intensive care unit; HCT: hematocrit

Outcome	Transfusion	%	No transfusion	%
Intraoperative blood loss	17		27	
≥ 1L	16	94.12%	8	29.63%
< 1L	1	5.88%	19	70.37%
Intraoperative blood transfusion				
0	0	0.00%	27	100.00%
1-2 units	9	52.94%	0	0.00%
≥ 3 units	8	47.06%	0	0.00%
Postoperative blood transfusion				
0	10	58.82%	26	96.30%
1-2 units	1	5.88%	1	3.70%
≥ 3 units	6	35.29%	0	0.00%
Massive blood transfusion				
Yes	6	35.29%	0	0.00%
No	11	64.71%	27	100.00%
Postpartum hemorrhage				
Yes	5	29.41%	1	3.70%
No	12	70.59%	26	96.30%
Hysterectomy				
Yes	6	35.29%	2	7.41%
No	11	64.71%	25	92.59%
ICU admission				
Yes	6	35.29%	0	0.00%
No	11	64.71%	27	100.00%
Length of stay in the ICU				
None	11	64.71%	27	100.00%
1-2 days	1	5.88%	0	0.00%
3-4 days	3	17.65%	0	0.00%
≥ 5 days	1	5.88%	0	0.00%
Not documented	1	5.88%	0	0.00%
Postoperative hemoglobin				
≤ 10	12	70.59%	14	51.85%
>10	5	29.41%	13	48.15%
Postoperative anemia (HCT)				
< 33% (Yes)	14	82.35%	20	74.07%
≥ 33% (No)	3	17.65%	7	25.93%
Postoperative thrombocytopenia				
< 150 (Yes)	12	70.59%	7	25.93%
≥ 150 (No)	5	29.41%	20	74.07%
APGAR 1 min				
≥ 7	6	35.29%	10	37.04%
4-6	8	47.06%	12	44.44%
≤ 3	0	0.00%	0	0.00%
Not documented	3	17.65%	5	18.52%
APGAR 5 min				
≥ 7	13	76.47%	20	74.07%
4-6	1	5.88%	2	7.41%
≤ 3	0	0.00%	0	0.00%
Not documented	3	17.65%	5	18.52%

## Discussion

The current case-control study was able to determine multiple statically significant risk factors associated with intraoperative blood transfusion for cesarean section in pregnancies with abnormal placentation. These factors include general anesthesia, placenta accreta, low pre-operative hematocrit (hematocrit < 33%), low preoperative hemoglobin (hemoglobin ≤ 10 g/dL), and preterm delivery at 35-36 weeks of gestation.

Boyle et al. reported no difference between general and regional anesthesia in bleeding outcomes in cesarean sections of previa patients [11]. However, many studies, including ours, have found a significant association between general anesthesia and increased intraoperative bleeding. Gibbins et al. concluded that general anesthesia was an independent risk factor of hemorrhagic morbidity in their cohort of previa patients [12]. Similarly, Hong et al. reported significantly increased transfusion requirements in patients given general anesthesia in cesarean sections for high-grade placenta previa [13]. One article has alluded that decreased uterine tone during general anesthesia is a possible explanation of this relation [14].

Multiple studies have indicated similar predicting factors as the present study. Titapant et al. implied that preoperative anemia (hematocrit < 33%) is an independent predictor of the need for intra-operative blood transfusion [14]. Similarly, Gibbins et al. reported that anemia on admission was linked to maternal hemorrhagic morbidity [12]. Additionally, Boyle et al. reported prematurity (gestational age of 32-35) as a predicting factor of intra-operative blood transfusion [11]. 

The present study has also compared operative and delivery outcomes in both groups. It was found that the transfusion group had more incidence of postpartum hemorrhage, hysterectomy, and ICU admissions. In contrast, no significant differences were found in the APGAR scores between the two groups. These results are comparable to those of previous studies. Boyle and colleagues found a significant correlation between transfusion requirements and hysterectomy levels [11]. However, in their study, hysterectomy was regarded as a risk factor for increased blood transfusion requirements and not as an outcome. Moreover, Titapant et al. reported comparable APGAR scores between their transfusion and non-transfusion groups of placenta previa patients [14]. Additionally, the scarcity of data on this topic is noted. Only a few studies have discussed the relation between blood transfusion requirements and postoperative complications in this group of patients.

The results of this study could enable better identification of patients at increased risk of bleeding. Thus, enhancing pre-operative preparation, counseling, intraoperative management, and prevention of related adverse outcomes.

Limitations and recommendations

One limitation of this study is the small sample size. Although 110 patients were available at the start of the data collection process, more than half of the patients had to be excluded, and only 44 patients were enrolled in the final dataset. This is attributed to the lack of necessary operative data in their electronic records. As a result, this study could be underpowered, and generalizability of the results would be limited. Additionally, only a correlation between factors and transfusion frequency could be inferred through our analysis. When logistic regression analysis was applied, none of the factors had enough statistical significance to be considered an independent predicting factor. This could also be attributed to the small sample size, in which factors would have been under-represented compared to their actual prevalence in the population.

The retrospective nature of the study, and the data available at our institution, are some other limitations. Many variables of interest could not be studied. Exploring sonographic features of previa in relation to bleeding risk was an interesting prospect, especially since Wang et al. found some interesting features that correlated significantly with bleeding risk [15]. Reproduction of a similar analysis was not feasible at our institution, as ultrasonic images were not available in patients’ electronic records. Similarly, details such as classification of previa and placental position were not recorded in many patients’ files, and thus these variables were not studied. Moreover, documentation of patients’ data was done through multiple physicians. Diagnosing and interpretation of ultrasound images, blood loss estimation, and decisions to transfuse blood products were made by different obstetricians and anesthesiologists, and thus the data could be operator-dependent.

It is possible to extend this research into a multi-center study; this would enhance the power of the study and make its results more generalizable. Moreover, a systematic review of similar studies could create a common consensus on the important factors that contribute to intraoperative bleeding. This would set the grounds for future guidelines to better recognize and treat patients who are at increased risk of bleeding.

## Conclusions

Abnormal placentation is associated with increased risk of intraoperative hemorrhage and blood transfusion. The need of intraoperative blood transfusion could be predicted if several factors are present. These factors include general anesthesia, placenta accreta, delivery at 35-36 weeks of gestation, and preoperative anemia. Identifying patients at increased risk is necessary to optimize perioperative and intraoperative management. This would aid in preventing associated feto-maternal complications, improving prognosis, and lowering associated morbidity and mortality rates.
